# 3-(4-Bromo­phen­yl)-5-[4-(dimethyl­amino)­phen­yl]-4,5-dihydro-1*H*-pyrazole-1-carbothio­amide

**DOI:** 10.1107/S1600536811006106

**Published:** 2011-02-23

**Authors:** Hoong-Kun Fun, Thitipone Suwunwong, Suchada Chantrapromma

**Affiliations:** a X-ray Crystallography Unit, School of Physics, Universiti Sains Malaysia, 11800 USM, Penang, Malaysia; b Crystal Materials Research Unit, Department of Chemistry, Faculty of Science, Prince of Songkla University, Hat-Yai, Songkhla 90112, Thailand

## Abstract

The mol­ecule of the title pyrazole derivative, C_18_H_19_BrN_4_S, is twisted. The central pyrazole ring, which adopts a flattened envelope conformation, is almost coplanar with the 4-bromo­phenyl ring, whereas it is inclined to the 4-(dimethyl­amino)­phenyl ring making dihedral angles of 1.68 (6) and 85.12 (6)°, respectively. The dihedral angle between the two benzene rings is 86.56 (6)°. The dimethyl­amino group is slightly twisted from the attached benzene ring [C—C—N—C torsion angles = 8.4 (2) and 8.9 (2)°]. In the crystal, mol­ecules are linked by inter­molecular N—H⋯S hydrogen bonds into chains along [2
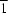
0]. The crystal is further stabilized by C—H⋯π inter­actions.

## Related literature

For background to chalcone synthesis and the biological activity of pyrazole derivatives, see: Bekhit *et al.* (2008 ▶); Ono *et al.* (2007 ▶); Cottineau *et al.* (2002 ▶); Gadakh *et al.* (2010 ▶); Hall *et al.* (2008 ▶); Hoepping *et al.* (2007 ▶); Mikhaylichenko *et al.* (2009 ▶); Park *et al.* (2005 ▶) Souza *et al.* (2002 ▶); Xie *et al.* (2008 ▶). For related structures, see; Chantrapromma *et al.* (2009 ▶); Suwunwong *et al.* (2009 ▶). For the stability of the temperature controller used in the data collection, see Cosier & Glazer (1986 ▶). For bond-length data, see: Allen *et al.* (1987 ▶). For puckering parameters, see: Cremer & Pople (1975 ▶).
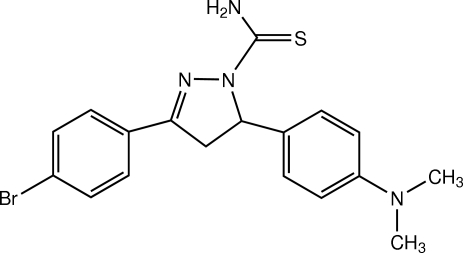


## Experimental

### 

#### Crystal data

C_18_H_19_BrN_4_S
*M* *_r_* = 403.34
Triclinic, 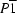

*a* = 6.9153 (1) Å
*b* = 9.5122 (1) Å
*c* = 15.1545 (2) Å
α = 72.196 (1)°
β = 80.941 (1)°
γ = 69.845 (1)°
*V* = 889.48 (2) Å^3^
*Z* = 2
Mo *K*α radiation
μ = 2.44 mm^−1^
*T* = 100 K
0.55 × 0.32 × 0.31 mm

#### Data collection

Bruker APEXII CCD area-detector diffractometer
Absorption correction: multi-scan (*SADABS*; Bruker, 2005 ▶) *T*_min_ = 0.349, *T*_max_ = 0.520
28456 measured reflections
7823 independent reflections
6784 reflections with *I* > 2σ(*I*)
*R*_int_ = 0.023

#### Refinement

*R*[*F* ^2^ > 2σ(*F* ^2^)] = 0.028
*wR*(*F* ^2^) = 0.073
*S* = 1.05
7823 reflections
227 parameters
H atoms treated by a mixture of independent and constrained refinement
Δρ_max_ = 0.96 e Å^−3^
Δρ_min_ = −0.50 e Å^−3^

### 

Data collection: *APEX2* (Bruker, 2005 ▶); cell refinement: *SAINT* (Bruker, 2005 ▶); data reduction: *SAINT*; program(s) used to solve structure: *SHELXTL* (Sheldrick, 2008 ▶); program(s) used to refine structure: *SHELXTL*; molecular graphics: *SHELXTL*; software used to prepare material for publication: *SHELXTL* and *PLATON* (Spek, 2009 ▶).

## Supplementary Material

Crystal structure: contains datablocks global, I. DOI: 10.1107/S1600536811006106/rz2558sup1.cif

Structure factors: contains datablocks I. DOI: 10.1107/S1600536811006106/rz2558Isup2.hkl

Additional supplementary materials:  crystallographic information; 3D view; checkCIF report

## Figures and Tables

**Table 1:** Hydrogen-bond geometry (Å, °) *Cg*1 and *Cg*2 are the centroids of the C1–C6 and C10–C15 rings, respectively.

*D*—H⋯*A*	*D*—H	H⋯*A*	*D*⋯*A*	*D*—H⋯*A*
N4—H1*N*4⋯S1^i^	0.84 (2)	2.54 (2)	3.3679 (11)	170.8 (17)
C5—H5*A*⋯*Cg*2^ii^	0.93	2.72	3.5462 (12)	149
C16—H16*B*⋯*Cg*2^iii^	0.96	2.71	3.6676 (18)	154
C17—H17*C*⋯*Cg*1^iv^	0.96	2.74	3.5990 (19)	149

Symmetry codes: (i) 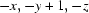; (ii) 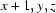; (iii) 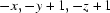; (iv) 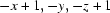.

**Fig. 1.**
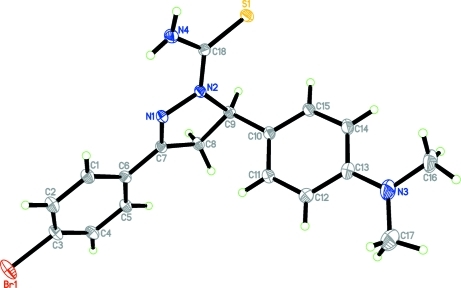
The molecular structure of the title compound, showing 50% probability displacement ellipsoids.

**Fig. 2.**
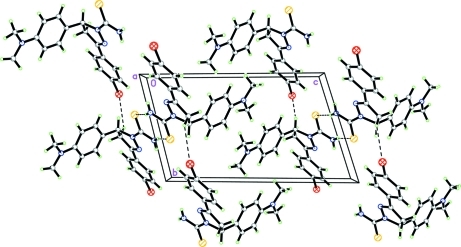
The crystal packing of the title compound viewed along the a axis. Hydrogen bonds are drawn as dashed lines.

## supplementary crystallographic information

### Comment

The pyrazole moiety is one of the core structures in a number of natural products (Xie *et al.*, 2008). Numerous compounds which contain the pyrazole moiety are known to exhibit a wide range of biological properties such as antihypertensive (Mikhaylichenko *et al.*, 2009), analgesic (Hall *et al.*, 2008), anti-inflammatory (Bekhit  *et al.*, 2008), antipyretic (Souza *et al.*, 2002), antimicrobial (Gadakh *et al.*, 2010), hypoglycemic (Cottineau *et al.*, 2002), sedative-hypnotic (Hoepping *et al.*, 2007) and antitumor activities (Park *et al.*, 2005). Our on going research on biological activities of pyrazole derivatives led us to synthesize the title compound by cyclization of the chalcone derivative (Ono *et al.*, 2007) with excess thiosemicarbazide. Herein we report the crystal structure of the title compound.

The molecular structure of the title compound is twisted. The central pyrazole ring adopts a flattened envelope conformation with puckering parameter Q = 0.1775 (11) Å and φ = 75.9 (3)° (Cremer & Pople, 1975), with the slightly puckered C9 atom having the maximum deviation of 0.1120 (11) Å. The pyrazole ring is coplanar with the 4-bromophenyl whereas inclined to the 4-dimethylaminophenyl rings with dihedral angles of 1.68 (6) and 85.12 (6)°, respectively. The dihedral angle between the two phenyl rings being 86.56 (6)°. The dimethylamino group is slightly twisted from the attached benzene ring with the torsion angles C16–N3–C13–C14 = 8.9 (2)° and C17–N3–C13–C12 = 8.4 (2)°. The carbothioamide is slightly twisted from the pyrazole ring as indicated by the torsions angles N4–C18–N2–N1 = 5.51 (14)° and S1–C18–N2–N1 = -172.28 (7)°. The bond distances agree with the literature values (Allen *et al.*, 1987) and are comparable to those observed in related structures (Chantrapromma *et al.*, 2009; Suwunwong *et al.*, 2009).

In the crystal structure (Fig. 2), the molecules are linked by intermolecular N—H···S hydrogen bonds (Table 1) into chains along the [2 1 0] direction. The crystal is further stabilized by C—H···π interactions (Table 1).

### Experimental

The title compound was synthesized by dissolving (*E*)-1-(4-bromophenyl)-3-(4-(dimethylamino)phenyl)prop-2-en-1-one (Ono *et al.*, 2007) (0.33 g, 1.0 mmol) in a solution of KOH (0.06 g, 1.0 mmol) in ethanol (20 ml). An excess thiosemicarbazide (0.14 g, 1.5 mmol) in ethanol (20 ml) was then added, and the reaction mixture was vigorously stirred and refluxed for 7 h. The yellow solid of the title compound obtained after cooling of the reaction mixture was filtered off under vacuum. Pale yellow block-shaped single crystals of the title compound suitable for *X*-ray structure determination were recrystalized from acetone/ethanol (1:1 *v*/*v*) by slow evaporation of the solvent at room temperature after several days. M.p. 481–482 K.

### Refinement

The amino H atoms were located in difference Fourier map and refined isotropically. The remaining H atoms were positioned geometrically and allowed to ride on their parent atoms, with d(C—H) = 0.93 Å for aromatic, 0.97 Å for CH_2_ and 0.96 Å for CH_3_ atoms. The *U*_iso_ values were constrained to be 1.5*U*_eq_ of the carrier atom for methyl H atoms and 1.2*U*_eq_ for the remaining H atoms. A rotating group model was used for the methyl groups. The highest residual electron density peak is located at 0.75 Å from Br1 and the deepest hole is located at 0.56 Å from Br1.

### Crystal data

**Table:**

C_18_H_19_BrN_4_S	*Z* = 2
*M**_r_* = 403.34	*F*(000) = 412
Triclinic, *P*1	*D*_x_ = 1.506 Mg m^−^^3^
Hall symbol: -P 1	Melting point = 481–482 K
*a* = 6.9153 (1) Å	Mo *K*α radiation, λ = 0.71073 Å
*b* = 9.5122 (1) Å	Cell parameters from 7823 reflections
*c* = 15.1545 (2) Å	θ = 2.4–35.1°
α = 72.196 (1)°	µ = 2.44 mm^−^^1^
β = 80.941 (1)°	*T* = 100 K
γ = 69.845 (1)°	Block, pale yellow
*V* = 889.48 (2) Å^3^	0.55 × 0.32 × 0.31 mm

### Data collection

**Table:**

Bruker APEXII CCD area-detector diffractometer	7823 independent reflections
Radiation source: sealed tube	6784 reflections with *I* > 2σ(*I*)
graphite	*R*_int_ = 0.023
φ and ω scans	θ_max_ = 35.1°, θ_min_ = 2.4°
Absorption correction: multi-scan (*SADABS*; Bruker, 2005)	*h* = −11→10
*T*_min_ = 0.349, *T*_max_ = 0.520	*k* = −15→15
28456 measured reflections	*l* = −24→24

### Refinement

**Table:**

Refinement on *F*^2^	Primary atom site location: structure-invariant direct methods
Least-squares matrix: full	Secondary atom site location: difference Fourier map
*R*[*F*^2^ > 2σ(*F*^2^)] = 0.028	Hydrogen site location: inferred from neighbouring sites
*wR*(*F*^2^) = 0.073	H atoms treated by a mixture of independent and constrained refinement
*S* = 1.05	*w* = 1/[σ^2^(*F*_o_^2^) + (0.0344*P*)^2^ + 0.3232*P*] where *P* = (*F*_o_^2^ + 2*F*_c_^2^)/3
7823 reflections	(Δ/σ)_max_ = 0.003
227 parameters	Δρ_max_ = 0.96 e Å^−^^3^
0 restraints	Δρ_min_ = −0.50 e Å^−^^3^

### Special details

**Table:**

Experimental. The crystal was placed in the cold stream of an Oxford Cryosystems Cobra open-flow nitrogen cryostat (Cosier & Glazer, 1986) operating at 100.0 (1) K.
Geometry. All e.s.d.'s (except the e.s.d. in the dihedral angle between two l.s. planes) are estimated using the full covariance matrix. The cell e.s.d.'s are taken into account individually in the estimation of e.s.d.'s in distances, angles and torsion angles; correlations between e.s.d.'s in cell parameters are only used when they are defined by crystal symmetry. An approximate (isotropic) treatment of cell e.s.d.'s is used for estimating e.s.d.'s involving l.s. planes.
Refinement. Refinement of *F*^2^ against ALL reflections. The weighted *R*-factor *wR* and goodness of fit *S* are based on *F*^2^, conventional *R*-factors *R* are based on *F*, with *F* set to zero for negative *F*^2^. The threshold expression of *F*^2^ > σ(*F*^2^) is used only for calculating *R*-factors(gt) *etc*. and is not relevant to the choice of reflections for refinement. *R*-factors based on *F*^2^ are statistically about twice as large as those based on *F*, and *R*- factors based on ALL data will be even larger.

### Fractional atomic coordinates and isotropic or equivalent isotropic displacement parameters (Å^2^)

**Table:**

	*x*	*y*	*z*	*U*_iso_*/*U*_eq_	
Br1	1.641401 (17)	−0.173962 (13)	0.169443 (10)	0.02713 (4)	
S1	0.08981 (4)	0.61386 (3)	0.083129 (18)	0.01668 (5)	
N1	0.62104 (13)	0.27982 (10)	0.13070 (6)	0.01465 (14)	
N2	0.44511 (13)	0.40189 (10)	0.14121 (6)	0.01404 (14)	
N3	−0.0497 (2)	0.25372 (15)	0.52890 (9)	0.0346 (3)	
N4	0.31575 (15)	0.37031 (12)	0.02111 (7)	0.01857 (16)	
C1	1.02110 (17)	0.05782 (12)	0.12650 (8)	0.01819 (18)	
H1A	0.9213	0.0544	0.0933	0.022*	
C2	1.21950 (17)	−0.04510 (13)	0.12460 (9)	0.02047 (19)	
H2A	1.2534	−0.1179	0.0908	0.025*	
C3	1.36736 (16)	−0.03752 (12)	0.17431 (8)	0.01850 (18)	
C4	1.32036 (16)	0.06853 (12)	0.22612 (8)	0.01758 (18)	
H4A	1.4208	0.0711	0.2593	0.021*	
C5	1.12052 (15)	0.17132 (12)	0.22782 (7)	0.01604 (17)	
H5A	1.0873	0.2432	0.2623	0.019*	
C6	0.96924 (15)	0.16730 (11)	0.17801 (7)	0.01418 (16)	
C7	0.76209 (15)	0.27851 (11)	0.17797 (7)	0.01395 (15)	
C8	0.69425 (15)	0.40713 (12)	0.22566 (7)	0.01553 (16)	
H8A	0.7585	0.4870	0.1952	0.019*	
H8B	0.7266	0.3671	0.2904	0.019*	
C9	0.45956 (15)	0.47007 (11)	0.21552 (7)	0.01428 (16)	
H9A	0.4135	0.5839	0.1937	0.017*	
C10	0.33157 (15)	0.41586 (12)	0.30181 (7)	0.01506 (16)	
C11	0.40552 (17)	0.27201 (13)	0.36660 (8)	0.01809 (18)	
H11A	0.5411	0.2104	0.3590	0.022*	
C12	0.28225 (18)	0.21843 (14)	0.44205 (8)	0.0221 (2)	
H12A	0.3371	0.1226	0.4842	0.027*	
C13	0.07487 (19)	0.30739 (14)	0.45555 (8)	0.0219 (2)	
C14	0.00187 (17)	0.45394 (14)	0.39134 (8)	0.01970 (19)	
H14A	−0.1332	0.5167	0.3988	0.024*	
C15	0.12813 (16)	0.50602 (13)	0.31725 (7)	0.01712 (17)	
H15A	0.0761	0.6039	0.2765	0.021*	
C16	−0.2680 (2)	0.3366 (2)	0.53468 (10)	0.0324 (3)	
H16A	−0.3276	0.3493	0.4785	0.049*	
H16B	−0.2880	0.4370	0.5428	0.049*	
H16C	−0.3334	0.2786	0.5866	0.049*	
C17	0.0361 (3)	0.1144 (2)	0.60089 (12)	0.0443 (4)	
H17A	0.1564	0.1203	0.6219	0.067*	
H17B	0.0737	0.0256	0.5768	0.067*	
H17C	−0.0645	0.1046	0.6519	0.067*	
C18	0.29283 (15)	0.45225 (12)	0.08212 (7)	0.01415 (16)	
H1N4	0.211 (3)	0.386 (2)	−0.0067 (13)	0.028 (4)*	
H2N4	0.414 (3)	0.287 (2)	0.0245 (12)	0.027 (4)*	

### Atomic displacement parameters (Å^2^)

**Table:**

	*U*^11^	*U*^22^	*U*^33^	*U*^12^	*U*^13^	*U*^23^
Br1	0.01316 (5)	0.01906 (5)	0.05023 (9)	−0.00079 (4)	−0.00226 (5)	−0.01560 (5)
S1	0.01295 (10)	0.01764 (10)	0.01829 (11)	−0.00210 (8)	−0.00309 (8)	−0.00530 (8)
N1	0.0112 (3)	0.0157 (3)	0.0161 (4)	−0.0029 (3)	−0.0005 (3)	−0.0046 (3)
N2	0.0112 (3)	0.0159 (3)	0.0149 (3)	−0.0024 (3)	−0.0014 (3)	−0.0059 (3)
N3	0.0276 (6)	0.0347 (6)	0.0316 (6)	−0.0095 (5)	0.0114 (5)	−0.0020 (5)
N4	0.0150 (4)	0.0224 (4)	0.0189 (4)	−0.0026 (3)	−0.0039 (3)	−0.0088 (3)
C1	0.0155 (4)	0.0180 (4)	0.0224 (5)	−0.0042 (3)	−0.0027 (3)	−0.0078 (4)
C2	0.0165 (4)	0.0178 (4)	0.0284 (5)	−0.0035 (3)	−0.0014 (4)	−0.0102 (4)
C3	0.0125 (4)	0.0140 (4)	0.0282 (5)	−0.0032 (3)	−0.0008 (3)	−0.0060 (4)
C4	0.0128 (4)	0.0158 (4)	0.0251 (5)	−0.0044 (3)	−0.0029 (3)	−0.0061 (4)
C5	0.0131 (4)	0.0150 (4)	0.0210 (4)	−0.0046 (3)	−0.0012 (3)	−0.0060 (3)
C6	0.0112 (4)	0.0140 (4)	0.0170 (4)	−0.0044 (3)	−0.0005 (3)	−0.0034 (3)
C7	0.0120 (4)	0.0142 (4)	0.0153 (4)	−0.0043 (3)	−0.0001 (3)	−0.0035 (3)
C8	0.0122 (4)	0.0167 (4)	0.0194 (4)	−0.0043 (3)	−0.0012 (3)	−0.0073 (3)
C9	0.0124 (4)	0.0153 (4)	0.0164 (4)	−0.0046 (3)	−0.0016 (3)	−0.0055 (3)
C10	0.0133 (4)	0.0168 (4)	0.0161 (4)	−0.0038 (3)	−0.0011 (3)	−0.0068 (3)
C11	0.0154 (4)	0.0189 (4)	0.0185 (4)	−0.0033 (3)	0.0000 (3)	−0.0059 (3)
C12	0.0207 (5)	0.0216 (5)	0.0194 (5)	−0.0046 (4)	0.0007 (4)	−0.0023 (4)
C13	0.0208 (5)	0.0256 (5)	0.0199 (5)	−0.0086 (4)	0.0040 (4)	−0.0078 (4)
C14	0.0147 (4)	0.0244 (5)	0.0204 (5)	−0.0041 (4)	0.0008 (3)	−0.0100 (4)
C15	0.0142 (4)	0.0195 (4)	0.0173 (4)	−0.0027 (3)	−0.0017 (3)	−0.0072 (3)
C16	0.0234 (6)	0.0522 (9)	0.0273 (6)	−0.0184 (6)	0.0087 (5)	−0.0162 (6)
C17	0.0419 (9)	0.0417 (8)	0.0351 (8)	−0.0142 (7)	0.0126 (7)	0.0035 (6)
C18	0.0124 (4)	0.0167 (4)	0.0131 (4)	−0.0051 (3)	−0.0005 (3)	−0.0032 (3)

### Geometric parameters (Å, °)

**Table:**

Br1—C3	1.8976 (10)	C7—C8	1.5091 (14)
S1—C18	1.6896 (10)	C8—C9	1.5391 (14)
N1—C7	1.2927 (13)	C8—H8A	0.9700
N1—N2	1.3901 (12)	C8—H8B	0.9700
N2—C18	1.3518 (13)	C9—C10	1.5155 (14)
N2—C9	1.4917 (13)	C9—H9A	0.9800
N3—C13	1.3759 (16)	C10—C11	1.3972 (15)
N3—C17	1.441 (2)	C10—C15	1.3990 (14)
N3—C16	1.4450 (19)	C11—C12	1.3893 (16)
N4—C18	1.3404 (14)	C11—H11A	0.9300
N4—H1N4	0.842 (19)	C12—C13	1.4113 (17)
N4—H2N4	0.841 (19)	C12—H12A	0.9300
C1—C2	1.3859 (15)	C13—C14	1.4090 (17)
C1—C6	1.4049 (15)	C14—C15	1.3848 (16)
C1—H1A	0.9300	C14—H14A	0.9300
C2—C3	1.3945 (16)	C15—H15A	0.9300
C2—H2A	0.9300	C16—H16A	0.9600
C3—C4	1.3848 (15)	C16—H16B	0.9600
C4—C5	1.3926 (14)	C16—H16C	0.9600
C4—H4A	0.9300	C17—H17A	0.9600
C5—C6	1.3999 (14)	C17—H17B	0.9600
C5—H5A	0.9300	C17—H17C	0.9600
C6—C7	1.4583 (14)		
			
C7—N1—N2	107.84 (8)	N2—C9—C8	100.12 (8)
C18—N2—N1	119.42 (8)	C10—C9—C8	114.94 (8)
C18—N2—C9	127.78 (8)	N2—C9—H9A	110.4
N1—N2—C9	112.61 (8)	C10—C9—H9A	110.4
C13—N3—C17	120.35 (12)	C8—C9—H9A	110.4
C13—N3—C16	120.36 (12)	C11—C10—C15	117.01 (10)
C17—N3—C16	119.29 (12)	C11—C10—C9	122.25 (9)
C18—N4—H1N4	117.0 (13)	C15—C10—C9	120.66 (9)
C18—N4—H2N4	120.0 (12)	C12—C11—C10	121.88 (10)
H1N4—N4—H2N4	119.5 (17)	C12—C11—H11A	119.1
C2—C1—C6	120.68 (10)	C10—C11—H11A	119.1
C2—C1—H1A	119.7	C11—C12—C13	120.90 (10)
C6—C1—H1A	119.7	C11—C12—H12A	119.6
C1—C2—C3	118.82 (10)	C13—C12—H12A	119.6
C1—C2—H2A	120.6	N3—C13—C14	121.54 (11)
C3—C2—H2A	120.6	N3—C13—C12	121.30 (11)
C4—C3—C2	121.77 (10)	C14—C13—C12	117.15 (10)
C4—C3—Br1	119.03 (8)	C15—C14—C13	121.01 (10)
C2—C3—Br1	119.19 (8)	C15—C14—H14A	119.5
C3—C4—C5	119.05 (10)	C13—C14—H14A	119.5
C3—C4—H4A	120.5	C14—C15—C10	122.00 (10)
C5—C4—H4A	120.5	C14—C15—H15A	119.0
C4—C5—C6	120.47 (10)	C10—C15—H15A	119.0
C4—C5—H5A	119.8	N3—C16—H16A	109.5
C6—C5—H5A	119.8	N3—C16—H16B	109.5
C5—C6—C1	119.21 (9)	H16A—C16—H16B	109.5
C5—C6—C7	120.05 (9)	N3—C16—H16C	109.5
C1—C6—C7	120.72 (9)	H16A—C16—H16C	109.5
N1—C7—C6	121.29 (9)	H16B—C16—H16C	109.5
N1—C7—C8	113.61 (8)	N3—C17—H17A	109.5
C6—C7—C8	124.98 (9)	N3—C17—H17B	109.5
C7—C8—C9	102.52 (8)	H17A—C17—H17B	109.5
C7—C8—H8A	111.3	N3—C17—H17C	109.5
C9—C8—H8A	111.3	H17A—C17—H17C	109.5
C7—C8—H8B	111.3	H17B—C17—H17C	109.5
C9—C8—H8B	111.3	N4—C18—N2	116.40 (9)
H8A—C8—H8B	109.2	N4—C18—S1	122.21 (8)
N2—C9—C10	110.17 (8)	N2—C18—S1	121.35 (8)
			
C7—N1—N2—C18	164.20 (9)	C7—C8—C9—N2	−16.35 (9)
C7—N1—N2—C9	−11.12 (11)	C7—C8—C9—C10	101.64 (9)
C6—C1—C2—C3	−0.38 (17)	N2—C9—C10—C11	82.45 (11)
C1—C2—C3—C4	0.78 (18)	C8—C9—C10—C11	−29.71 (13)
C1—C2—C3—Br1	−178.27 (9)	N2—C9—C10—C15	−94.19 (11)
C2—C3—C4—C5	−0.65 (17)	C8—C9—C10—C15	153.64 (9)
Br1—C3—C4—C5	178.40 (8)	C15—C10—C11—C12	1.39 (16)
C3—C4—C5—C6	0.12 (16)	C9—C10—C11—C12	−175.37 (10)
C4—C5—C6—C1	0.25 (16)	C10—C11—C12—C13	0.81 (18)
C4—C5—C6—C7	−178.15 (10)	C17—N3—C13—C14	−170.94 (15)
C2—C1—C6—C5	−0.12 (16)	C16—N3—C13—C14	8.9 (2)
C2—C1—C6—C7	178.27 (10)	C17—N3—C13—C12	8.4 (2)
N2—N1—C7—C6	−177.42 (9)	C16—N3—C13—C12	−171.79 (13)
N2—N1—C7—C8	−1.24 (11)	C11—C12—C13—N3	178.51 (13)
C5—C6—C7—N1	178.25 (10)	C11—C12—C13—C14	−2.16 (18)
C1—C6—C7—N1	−0.12 (15)	N3—C13—C14—C15	−179.34 (12)
C5—C6—C7—C8	2.53 (15)	C12—C13—C14—C15	1.33 (17)
C1—C6—C7—C8	−175.85 (10)	C13—C14—C15—C10	0.88 (17)
N1—C7—C8—C9	12.11 (11)	C11—C10—C15—C14	−2.23 (16)
C6—C7—C8—C9	−171.88 (9)	C9—C10—C15—C14	174.59 (10)
C18—N2—C9—C10	81.36 (12)	N1—N2—C18—N4	5.51 (14)
N1—N2—C9—C10	−103.80 (9)	C9—N2—C18—N4	−179.96 (9)
C18—N2—C9—C8	−157.18 (10)	N1—N2—C18—S1	−172.28 (7)
N1—N2—C9—C8	17.65 (10)	C9—N2—C18—S1	2.25 (15)

### Hydrogen-bond geometry (Å, °)

**Table:**

*Cg*1 and *Cg*2 are the centroids of the C1–C6 and C10–C15 rings, respectively.

**Table:**

*D*—H···*A*	*D*—H	H···*A*	*D*···*A*	*D*—H···*A*
N4—H1N4···S1^i^	0.84 (2)	2.54 (2)	3.3679 (11)	170.8 (17)
C5—H5A···Cg2^ii^	0.93	2.72	3.5462 (12)	149
C16—H16B···Cg2^iii^	0.96	2.71	3.6676 (18)	154
C17—H17C···Cg1^iv^	0.96	2.74	3.5990 (19)	149

Symmetry codes: (i) −*x*, −*y*+1, −*z*; (ii) *x*+1, *y*, *z*; (iii) −*x*, −*y*+1, −*z*+1; (iv) −*x*+1, −*y*, −*z*+1.
